# Network Analysis of Demographics, Dietary Intake, and Comorbidity Interactions

**DOI:** 10.3390/nu13103563

**Published:** 2021-10-12

**Authors:** Tung Hoang, Jeonghee Lee, Jeongseon Kim

**Affiliations:** Department of Cancer Biomedical Science, National Cancer Center Graduate School of Cancer Science and Policy, Goyang 10408, Korea; hoangtunghup@gmail.com (T.H.); jeonghee@ncc.re.kr (J.L.)

**Keywords:** network analysis, Gaussian graphical model, mixed graphical model

## Abstract

The aim of this study was to elucidate the complex interrelationships among dietary intake, demographics, and the risk of comorbidities. We applied a Gaussian graphical model to calculate the dietary scores of the participants. The network structure of dietary intake, demographics, and comorbidities was estimated in a mixed graphical model. The centrality indices of the nodes (strength (S), closeness (C), and betweenness (B)) were measured to identify the central node. Multinomial logistic regression was used to examine the association between the factors and comorbidities. Among 7423 participants, the strongest pairwise interactions were found between sex and smoking (1.56), sex and employment (0.66), sex and marital status (0.58), marital status and income (0.65), and age and employment (0.58). Among the factors in the network, sex played a central role (S = 4.63, C = 0.014, B = 41), followed by age (S = 2.81, C = 0.013, B = 18), smoking (S = 2.72, C = 0.013, B = 0), and employment (S = 2.17, C = 0.014, B = 22). While the odds of hypertension and diabetes were significantly higher among females than males, an inverse association was observed between high cholesterol and moderate chronic kidney disease. Among these factors, dietary intake was not a strongly interacting factor in the network, whereas age was consistently associated with the comorbidities of hypertension, high cholesterol, diabetes, and chronic kidney disease.

## 1. Introduction

Several studies have reported significant associations between comorbidities and demographic and dietary variables. For example, a recent comprehensive meta-analysis of 93 individual studies found that an unhealthy diet was associated with a higher body mass index (BMI), whereas a healthy diet was associated with a higher education level, greater physical activity, and reduced smoking behaviors [1]. Additional pooled individual-level data of American adults showed that obesity was significantly associated with a higher risk of cardiovascular disease (CVD) [2]. Moreover, other demographic factors, such as smoking tobacco and alcohol consumption, have been shown to result in several negative health consequences [3,4,5]. Regarding disease development, smoking is not only a risk factor itself but may also be associated with many socioeconomic factors, including income, educational level, and employment status [6,7]. Furthermore, accumulating evidence has indicated that dietary intake contributes to the risk of chronic diseases [8,9,10]. Julibert et al. recently reported a higher prevalence of metabolic syndrome—which is associated with CVD and type 2 diabetes mellitus [11]—among participants consuming higher amounts of total fat and lower amounts of carbohydrates and fiber in their diets [8]. Another network meta-analysis of 3595 participants showed a beneficial effect of nut intake on low-density lipoprotein cholesterol and triglycerides [12], supporting the negative association between nut intake and CVD risk [13].

The association between exposures and outcomes, such as the aforementioned associations, can be measured statistically using linear, logistic, Cox, and Poisson regression techniques [14]. Although variable selection approaches, such as stepwise, backward, and forward selection, may be sufficient to address many interdependencies among predictors in epidemiological data, it is difficult to demonstrate the accuracy of the obtained results [15]. These conventional approaches also have limitations in explaining complex relationships, such as biological pathways in systems epidemiology [16]. Additionally, due to the development of technologies, large-scale “omic” data sets of genome, transcriptome, proteome, metabolome, and microbiome data may include numerous variables [16,17]. In this case, a network analysis with graphical theory, which consists of nodes (vertices) that indicate factors and edges (links) that represent relationships, such as correlation coefficients among the factors, can provide insights into the interactions among all the variables and explore how a single variable is impacted by multiple factors [15,18]. In nutritional epidemiology, Iqbal et al. applied a Gaussian graphical model (GGM) to derive networks of dietary patterns in a German population, in which the partial correlations between two food groups conditioning the remaining food groups were estimated [19]. Solmi et al. recently applied a mixed graphical model (MGM) to investigate the interrelationship of various factors in a cohort of elderly adults at risk of osteoarthritis [20]. In contrast to the GGM, the MGM is able to identify a network structure of regularized interactions among both categorical and continuous variables. However, both dietary score and comorbidities in Solmi et al.’s study were measured using fixed scales, such as the Mediterranean dietary adherence score and the Charlson comorbidity index, which might not generalize to different populations [20].

Given that the complex associations among demographics, dietary behaviors, and comorbidities may provide valuable insight into disease development, we conducted this study to describe the interactions among these factors using an MGM. Additionally, we investigated the associations among demographics, dietary intake, and the risk of chronic diseases.

## 2. Materials and Methods

### 2.1. Data and Participants

This study used baseline survey data from the Cancer Screening Examination Cohort at the National Cancer Center (NCC), South Korea, from 16 October 2007 to 24 May 2019 (*n* = 16,188). Further details of this process were previously described [21]. Data from a structured questionnaire, clinical tests, physical functions, and blood tests were extracted for this study [21]. In addition, data on dietary behaviors were collected via a validated food frequency questionnaire (FFQ) with 106 food items [22,23].

**Figure 1:**
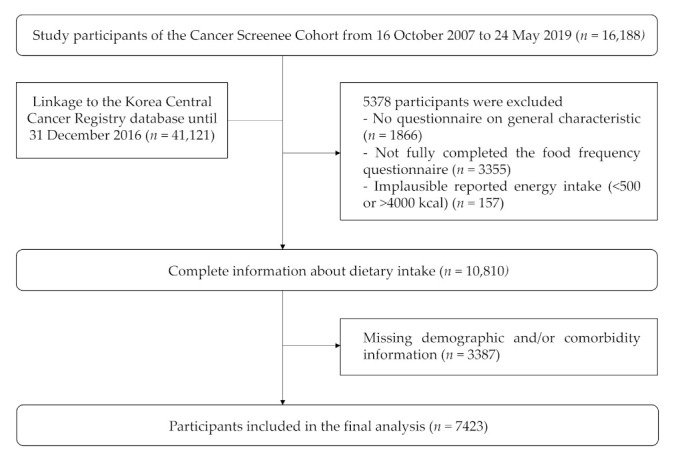
Flowchart of the recruitment and selection process.

The participants who did not complete the questionnaire that assessed general characteristics or the FFQ, and those who reported unrealistic data for energy consumption (<500 or >4000 kcal) were excluded (*n* = 5378). Of the 10,810 remaining participants, 7423 participants were ultimately included in the final network analysis after removing those with missing values for demographic and comorbidity information (Figure 1). The study protocol was approved by the Institutional Review Board of the NCC (number NCCNCS-07-077).

### 2.2. Variable Measurements

The demographic variables included age (years), sex (male and female), marital status (married, cohabitation, and others), education (<high school, high school graduate, and ≥college), monthly income (<2 million, 2–4 million, and ≥4 million KRW), smoking status (never, past, and current), alcohol consumption (never, past, and current), and regular exercise (yes and no). Although the World Health Organization recommends the standard BMI levels for underweight, normal, overweight, and obese individuals (i.e., <18.5, 18.5–24.9, 25–29.9, and ≥30 kg/m^2^, respectively), the cutoff BMI for the Korean population was identified as 23 kg/m^2^ and 25 kg/m^2^, considering the higher body fat percentages in Asians than in non-Asians, and the increased risks of any comorbidities, including diabetes, hypertension, and dyslipidemia [24,25]. Additionally, the participants in the standard underweight and obesity groups accounted for relatively small proportions of the total study population (approximately 2% each), with 175 (2.4%), 3216 (43.3%), 1978 (26.6%), 1870 (25.2%), and 184 (2.5%) participants with BMIs of <18.5, 18.5–22.9, 23–24.9, 25–29.9, and ≥30 kg/m^2^, respectively. Therefore, we selected the following cutoffs for the BMI for the final analysis: <23, 23–24.9, and ≥25 kg/m^2^.

The dietary intake (g/day) of 16 food groups was calculated using the Computer-Aided Nutritional Analysis Program (CAN-Pro) 4.0 (Computer-Aided Nutritional Analysis Program, The Korean Nutrition Society, Seoul, Korea). Then, these intakes were log-transformed, and their pairwise correlations were estimated in the GGM [19]. Weights according to food groups were obtained as the eigenvector centrality of the GGM-estimated network to compute the dietary intake score [26]. The dietary score was calculated as the sum of the amount of each food group consumed (g/day) by their respective weights. The higher dietary scores were categorized into tertiles, representing the higher GGM-weighted food consumption and implying the light, normal, and heavy eating behaviors.

Comorbid conditions were identified based on blood pressure, total cholesterol, fasting glucose, glomerular filtration rate (GFR) levels, and self-reported hypertension or diabetes. According to the Joint National Committee guidelines, blood pressure (BP) (mmHg) was classified as normal (diastolic blood pressure (DBP) < 80 and systolic blood pressure (SBP) < 120, without treatment, and no self-reported hypertension), elevated (DBP 80–89 or SBP 120–139, without treatment and no self-reported hypertension), and hypertension (DBP ≥ 90, SBP ≥ 140, treatment or self-reported hypertension) [27]. The total cholesterol (mmol/L) was classified as <4.66 (optimal), 4.67–5.18 (normal range for people without heart diseases or diabetes), 5.19–6.21 (borderline high), and ≥6.22 (high risk) [28]. The World Health Organization recommends defining the fasting glucose level (mmol/L) as normal (<6.11, without treatment, and no self-reported diabetes), prediabetic, and diabetic (≥6.11 or with treatment or self-reported diabetes) [29]. GFR (mL/min/1.73 m^2^), which was calculated by the Modification of Diet in Renal Disease equation, was divided into groups of ≥90 (normal), 60–89 (mildly impaired kidney function), and <60 (moderately impaired kidney function) [30].

### 2.3. Network Analysis

The network analysis of the demographics, dietary intake, and comorbidity factors was performed using an MGM, in which nodes reflect both categorical and continuous variables, and edges reflect their pairwise interactions [31]. In particular, we applied the “mgm” package, which was developed by Haslbeck and Waldorp, to obtain the network estimation of time-varying k-order MGMs [31]. Lasso regularization with extended Bayesian information criteria (EBIC) model selection, which is considered to be more conservative and have slightly higher precision than the cross-validation procedure, was applied and set at 0.5 to estimate the network structures [32,33,34]. The parameter of the interaction between two continuous variables indicates their partial correlation after controlling for the remaining variables [31]. In the case of a continuous variable and a categorical variable, the parameter of the interaction indicates the relationship between the continuous variable and the probability of observing category 1 of the categorical variable [31]. The parameter between two categorical variables corresponds to the interaction between two corresponding indicator variables [31]. When combining the coefficients estimated by the nodewise regression procedure into one edge parameter, all the estimates are required to be nonzero [31].

Regarding the importance of the nodes, we assessed the centrality indices, including strength (S; how well a node is directly connected to the other nodes), closeness (C; how well a node is indirectly connected to the other nodes), and betweenness (B; how important a node is in the mediation between two other nodes) [35,36]. The network accuracy was assessed by bootstrapping 80% of the original sample with a replacement [36,37].

In the sensitivity analysis, we additionally constructed an MGM-identified network, including 16 food groups instead of the dietary score. We also considered the interactions among the remaining variables after excluding nonmodifiable factors, such as age and sex.

### 2.4. Association Analysis

The statistical differences among the comorbidity statuses according to the demographic factors and dietary intakes were assessed using an ANOVA test and *t*-test (for continuous variables) or a Chi-square test (for the categorical variables). Multiple and multinomial logistic regressions were used to explore the associations of demographics and dietary intake with comorbidities.

All statistical analyses were performed in R version 3.6.0 (Foundation for Statistical Computing, Vienna, Austria).

## 3. Results

### 3.1. Dietary Score Measurements

**Figure 2:**
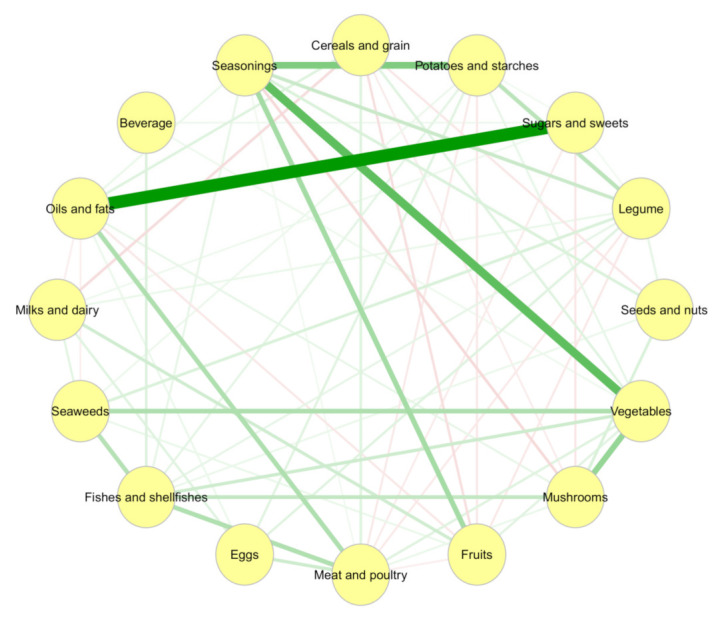
Network of dietary intake derived by Gaussian graphical models. Nodes reflect food groups, and edges reflect the conditional dependencies between food groups. Green lines show positive partial correlations, and red lines show negative partial correlations. The thickness of edges represents the strength of the correlations.

The GGM identified the dietary intake network (Figure 2), and the adjacency matrix of the regularized partial correlation is shown in Table S1. Sugars and sweets were observed to exhibit the strongest partial correlation with oils and fats (0.67), followed by seasonings with vegetables (0.42) and potatoes with starches (0.33). The negative partial correlations weakly ranged from −0.10 to −0.04. The personalized dietary scores were calculated based on the eigenvector centrality of these edge weights and the raw dietary intake amount (g/day) of the node sizes. As a result, the dietary score of the whole study population was 592.4 ± 308.6.

### 3.2. Characteristics of Study Participants

The characteristics of the study participants are presented in Table 1, Table 2, Table 3 and Table 4. Age, employment status, smoking status, alcohol consumption, and BMI significantly differed among the BP, total cholesterol, fasting glucose, and GFR comorbidity marker groups (*p* < 0.05). The other demographic factors and the intake of 16 food groups were equally distributed under at least one comorbidity condition. Additionally, the GGM-identified dietary scores were classified into low, medium, and high quantiles, and variations among the BP and GFR groups were observed (*p* ≤ 0.01).

### 3.3. Network Structure

**Figure 3:**
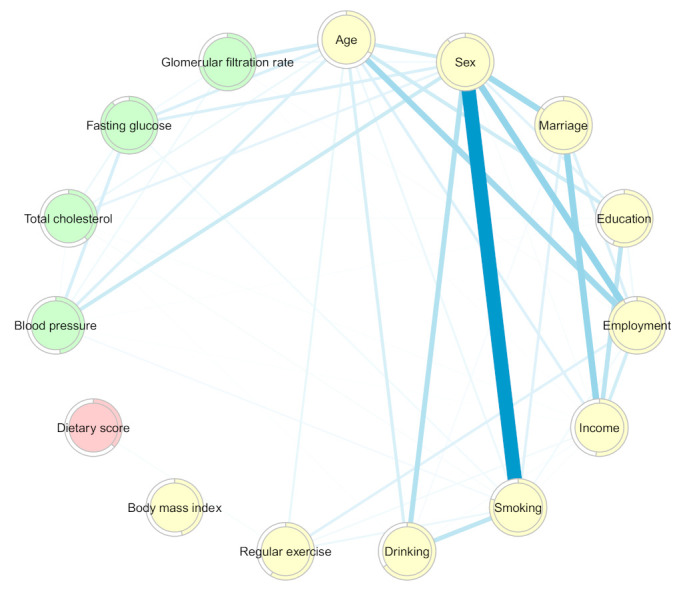
Network of dietary intake, demographics, and metabolic markers derived from mixed graphical models. Nodes reflect dietary score (pink), demographic factors (yellow), and metabolic markers (green), and edges reflect the pairwise interactions between variables. The thickness of the blue edges represents the strength of the interactions. Colored rings are proportional to prediction functions, including explained variance for continuous variables and correct classification for categorical variables.

The MGM-identified network structure of the pairwise interactions among the dietary score, demographics, and comorbidity markers is shown in Figure 3, and the weighted adjacency matrix is presented in Table S2. In general, all the factors were pairwise related to each other, except for BMI, which was independent of the network of interactions. Regarding dietary intake, an interaction was found only with regular exercise (0.05).

Age was found to have the greatest interaction with employment (0.58), followed by sex (0.33), education (0.31), GLR (0.30), alcohol consumption (0.25), BP (0.24), fasting glucose (0.23), income (0.21), and smoking and total cholesterol (0.12).

The strongest interactions were observed between sex and smoking (1.56), employment (0.66), marital status (0.58), smoking (0.47), age (0.33), BP (0.32), and fasting glucose level (0.23). Sex also interacted with education (0.19), total cholesterol (0.17), GLR (0.08), and income (0.05).

Marital status was observed to interact with income (0.65), sex (0.58), and employment (0.23) and was slightly related to alcohol consumption (0.06). There was an interaction between educational level and income (0.42), age (0.31), sex (0.19), and employment (0.10). Educational level was also found to be slightly related to total cholesterol (0.03) and BP (0.02).

While monthly income interacted strongly with marital status (0.65), education (0.42), employment (0.28), and age (0.21), slight interactions of monthly income with sex, smoking, alcohol consumption, exercise, BP, total cholesterol, and GFR were observed (<0.10).

Interactions of tobacco smoking and alcohol consumption with age, sex, marital status, income, and total cholesterol were found. Smoking additionally interacted with exercise (0.12), fasting glucose (0.09), employment (0.08), and BP (0.07).

There were slight interactions between regular exercise and age (0.14), smoking (0.12), income (0.08), and dietary score (0.05).

Regarding comorbidities, the interaction weights between chronic disease markers and demographic factors ranged from 0.02 to 0.32, and approximately half of the interaction weights were lower than 0.10.

### 3.4. Network Inference

**Figure 4:**
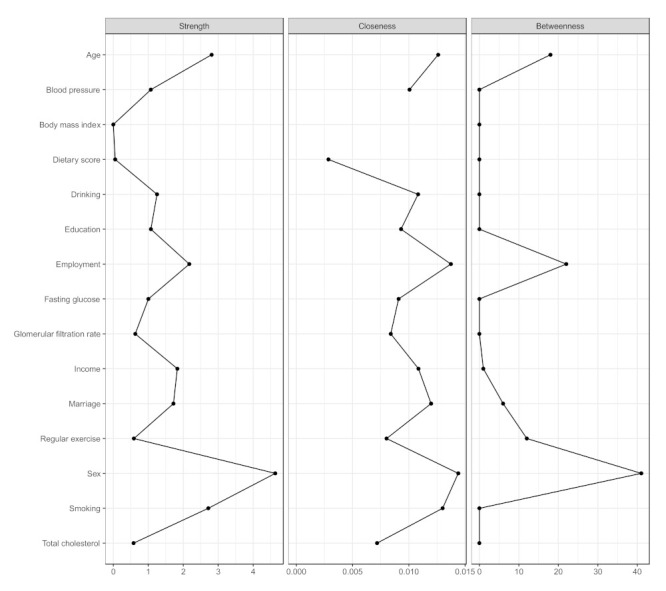
Centrality indices of the network of dietary intake, demographics, and metabolic markers.

The centrality indices are presented in Figure 4 and Table S3. In the network of dietary intake, demographics, and comorbidities, sex was the most central node (S = 4.63, C = 0.014, B = 41), followed by age (S = 2.81, C = 0.013, B = 18), smoking (S = 2.72, C = 0.013, B = 0), and employment (S = 2.17, C = 0.014, B = 22).

### 3.5. Network Stability

The accuracy of the network inference was investigated by nonparametric bootstrapping. The variability in the edge weights is shown in Figure S1; many bootstrapped confidence intervals (CIs), which were sufficiently small, suggested the high accuracy of the estimated edge weights. Additionally, the edges between sex and smoking and between sex and marriage were suggested as the two strongest edges because their bootstrapped CIs did not overlap with the bootstrapped CIs of any other edges. Figure S2 shows the stability of the centrality indices in a subset of the data. Although the node strength seemed to be stable and the node betweenness tended to gradually decrease, the CS coefficients were still high, with values of 0.75 and 0.60. In this case, node closeness was not appropriate since BMI was independent of the other nodes. The significant differences in the edge weights and node strengths are presented in Figures S3 and S4. Generally, approximately half of the edge weights did not differ from one another (Figure S3), while most of the node strengths significantly differed from one another (Figure S4).

### 3.6. Sensitivity Analysis

**Figure 5:**
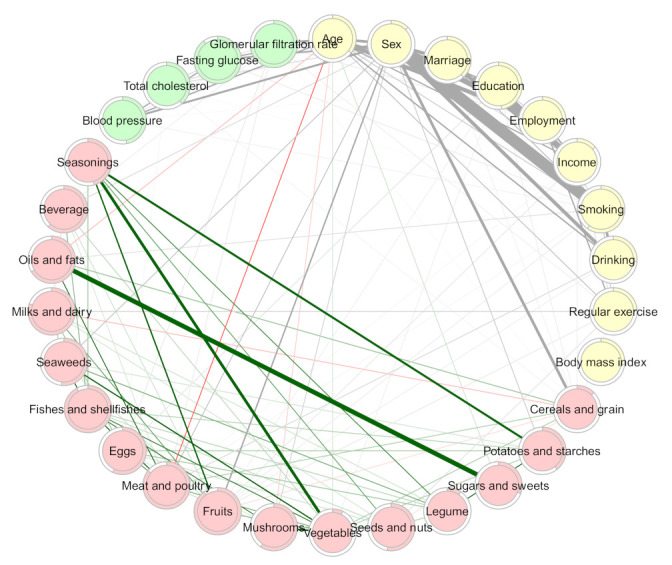
Network of food groups, demographics, and metabolic markers derived by mixed graphical models. Nodes reflect food groups (pink), demographic factors (yellow), and metabolic markers (green), and edges reflect the pairwise interactions between variables. The thickness of the blue edges represents the strength of the interactions. Colored rings are proportional to prediction functions, including explained variance for continuous variables and correct classification for categorical variables.

**Figure 6:**
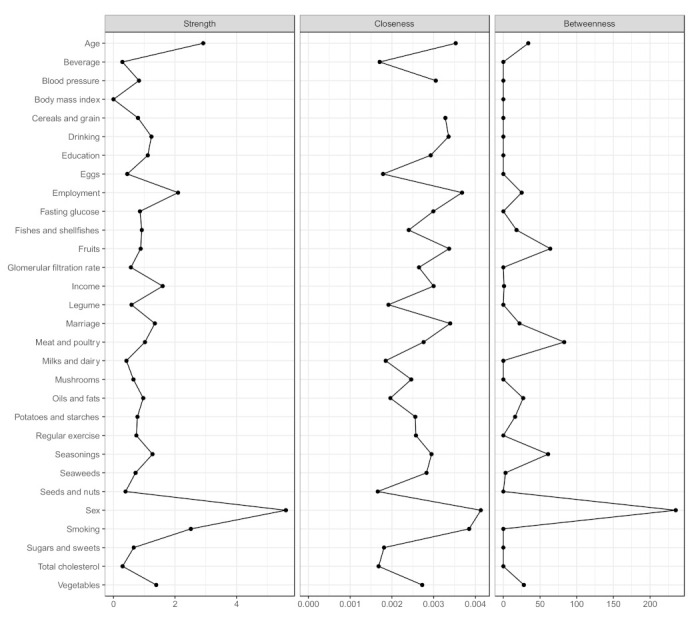
Centrality indices of the network of food groups, demographics, and metabolic markers.

The MGM-identified network, when including single food groups instead of the dietary score, is presented in Figure 5 and Figure 6 and Tables S4 and S5. Overall, the food groups slightly interacted with the demographic factors and comorbidities (weights ranged between 0.01 and 0.12), except for cereals and grains and sex (0.40), and fruits and sex (0.23). Sex was still the most central node (S = 5.60, C = 0.004, B = 235).

**Figure 7:**
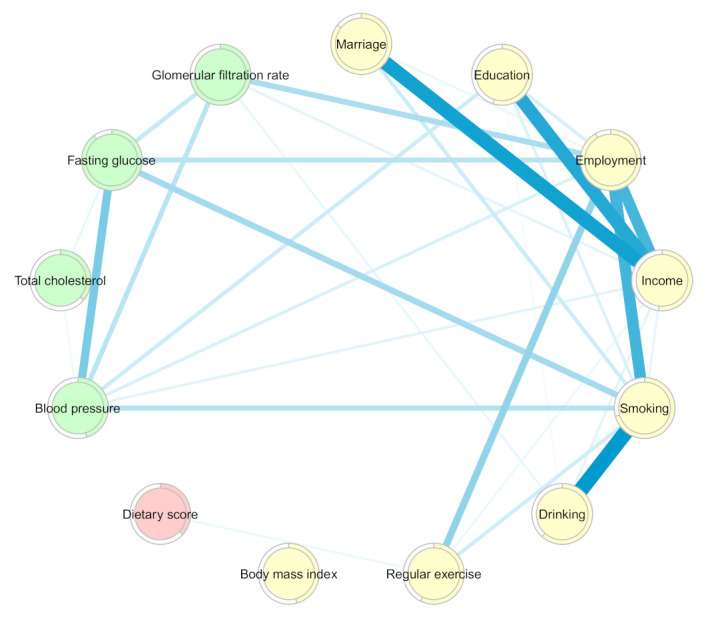
Network of food groups, modifiable demographics, and metabolic markers derived by mixed graphical models. Nodes reflect food groups (pink), modifiable demographic factors (yellow), and metabolic markers (green), and edges reflect the pairwise interactions between variables. The thickness of the blue edges represents the strength of the interactions. Colored rings are proportional to prediction functions, including correct classification for categorical variables.

**Figure 8:**
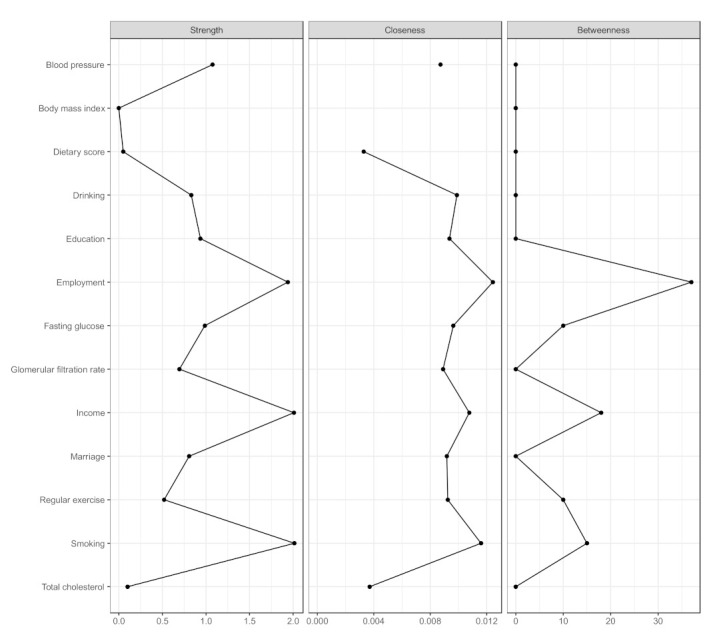
Centrality indices of the network of food groups, modifiable demographics, and metabolic markers.

In the sensitivity analysis constructed by removing age and sex from the MGM-identified network (Figure 7 and Figure 8 and Tables S6 and S7), the dietary score slightly interacted only with regular exercise (0.05); employment (S = 1.94, C = 0.012, B = 37), income (S = 2.01, C = 0.011, B = 18), and smoking (S = 2.01, C = 15) were central nodes.

### 3.7. Association among Demographics, Dietary Score, and Comorbidities

Table 5 shows the ORs and 95% CIs of the associations between demographic and dietary factors with comorbidities. Age was found to be associated with a higher odds of elevated BP (OR, 1.70; 95% CI, 1.41–2.04, *p* < 0.001) and hypertension (OR, 5.86; 95% CI, 4.80–7.16, *p* < 0.001), borderline (OR, 1.42; 95% CI, 1.17–1.72, *p* < 0.001) and high cholesterol (OR, 2.17; 95% CI, 1.67–2.82, *p* < 0.001), and prediabetes and diabetes (OR, 3.93; 95% CI, 3.08–5.00, *p* < 0.001). The effect of age on the risk of mildly impaired kidney function (OR, 1.21; 95% CI, 1.01–1.45, *p* = 0.04) and moderate chronic kidney disease (OR, 12.5; 95% CI, 8.28–19.0, *p* < 0.001) was observed only in the elderly participants (≥60 years). Females were much more likely to have elevated BP (OR, 2.52; 95% CI, 2.08–3.04, *p* < 0.001), hypertension (OR, 2.94; 95% CI, 2.38–3.63, *p* < 0.001), prediabetes and diabetes (OR, 1.79; 95% CI, 1.39–2.31, *p* < 0.001), but fewer females had borderline BP (OR, 0.78; 95% CI, 0.64–0.96, *p* = 0.02), high cholesterol (OR, 0.43; 95% CI, 0.32–0.57, *p* < 0.001), and moderate kidney impairment (OR, 0.52; 95% CI, 0.33–0.82, *p* = 0.01). The participants who were high school graduates showed higher odds of low, borderline, and high cholesterol. There was a significantly increased risk of prediabetes and diabetes in the smoking groups, including both current smokers (OR, 1.63; 95% CI, 1.22–2.18, *p* = 0.001) and past smokers (OR, 1.40; 95% CI, 1.09–1.81, *p* = 0.01). Additionally, smoking was similarly positively associated with high cholesterol (OR, 1.72; 95% CI, 1.24–2.40, *p* = 0.001) but negatively associated with elevated BP (OR, 0.60; 95% CI, 0.48–0.76, *p* < 0.001) and hypertension (OR, 0.75; 95% CI, 0.58–0.97, *p* = 0.03). Regarding dietary intake, normal, and heavy eating were significantly associated with increases of at least 20% in the risks of elevated BP, hypertension, and mild kidney impairment.

## 4. Discussion

Given that the complex correlations among dietary intake, demographics, and chronic diseases might contribute to the progression of other health conditions, we first described the pairwise interactions among age, sex, marital status, educational level, employment status, monthly income, smoking status, alcohol consumption, physical activity, BMI, dietary score, and chronic diseases related to BP, total cholesterol, fasting glucose, and GFR. The strongest pairwise interactions were found between sex and smoking, sex and employment, sex and marital status, marital status and income, and age and employment. Among the factors in the network, sex had a central role, followed by age, smoking, and employment. Second, we found that aging was a consistent risk factor associated with hypertension, high cholesterol, diabetes, and chronic kidney disease. While the odds of hypertension and diseases were significantly higher among females than males, the inverse association was observed in terms of high cholesterol and moderate chronic kidney disease.

Regarding the interaction between sex and smoking, Peters et al. recently investigated the sex dissimilarity in tobacco consumption in the UK Biobank study, which included approximately 500,000 participants. It was shown that differences in smoking habits have decreased over time in the Western population [38]. Several meta-analyses also reported the considerable risk of chronic diseases and cancer associated with smoking regardless of sex [39,40]. In a pooled meta-analysis that involved approximately one million Asian participants, although tobacco smoking among males accounted for approximately 60% of lung cancer mortality, the findings among females still varied according to country and region [41]. Nevertheless, the prevalence of a daily smoking habit was 25.0% among males, compared with 5.4% among females worldwide [42]. Similarly, the smoking prevalence was much higher in males (46.6% in 2005 and 42.3% in 2014) than in females (4.6% in 2005 and 5.1% in 2014) among Korean adults, which may support the strong interaction between sex and smoking status in our study [43,44].

In addition, the interaction between sex and employment also reflected an imbalance in employment rates between males (approximately 70%) and females (approximately 50%) during the 2007–2018 period in South Korea [45]. Although Korean women are highly skilled and educated, many of them are encouraged to discontinue permanent employment due to social expectations [46].

Furthermore, a high monthly income not only interacted with marital status but was also significantly associated with a decreased risk of marriage dissolution [47]. In East Asian countries, such as Korea, Japan, China, and Taiwan, this finding could be explained by the high financial cost of raising children as the main reason for delayed marriage [48].

Regarding factors associated with chronic diseases, age was determined to play an important role in disease pathology [49]. In the body, senescent cells are induced in normal aging, age-related disease, and therapeutic intervention contexts [49,50]. In aging people, senescence, which is normally limited to some organs and tissues, becomes dysfunctional due to aging, resulting in a much higher accumulation of senescent cells than in normal aging individuals and can cause age-related chronic diseases [49]. Moreover, senescent cells were reported to contribute to metabolic syndromes regulated by AMPK, GSK3, and mTOR-signaling kinases [51].

To the best of our knowledge, this study was the first in which the interactions among dietary intake, demographics, and comorbidity markers in the Korean population were investigated. In general, stationary assumption is defined as the stability of the mean, variance, and autocorrection of the data over time. Although all the information was recorded at the initial time of enrollment in the study, the time-varying model of MGM can reduce the assumption of stationary data such that the parameters are allowed to vary at the same time point, and thus, the results may represent the long-term relationship among the variables [52]. Several dietary score scales have been developed to assess a healthy diet [53]; however, such methods may not be appropriate due to natural differences in the dietary behaviors of different populations. In this study, we applied the data-driven approach of the GGM to generate an overall dietary score that represents the eating behavior of light eating, normal eating, and heavy eating for each individual based on the amount of food consumed and the eigenvector centrality of the identified network as weights. Giving weights to each node of a food group in the calculation of the dietary score may better estimate the eating behavior than just simply adding or subtracting the food group consumption when combining food groups in previous studies [54]. Furthermore, chronic comorbidities were identified based on not only self-reports but also clinical test results, which increased the accuracy of disease status identification.

Despite several strengths, this study has some limitations. Although the study recruited a large number of participants from a health screening program over a period of ten years, the cross-sectional study design may not have allowed for a full investigation of the causal relationship between the demographic and dietary factors and metabolic markers. Additionally, selection bias could have occurred due to the hospital-based setting; therefore, the results may not represent the entire population. When using structured questionnaires as tools to obtain information, recall of habit information can differ between males and females and between those with and without diseases because of different levels of health compliance. However, the validated and reproducible FFQ was administered by well-trained staff, which could have minimized the risk of collecting inaccurate information [21,23]. Furthermore, approximately 30% of the participants were excluded from the final analysis due to missing values that could not be accommodated. Finally, since the dietary score represents the overall dietary habits, it is difficult to interpret the role of specific food groups in the development of comorbidities.

## 5. Conclusions

In conclusion, this study investigated the comprehensive interaction network among dietary intake, demographics, and comorbidities in Korean adults. Among the factors studied, age, sex, smoking, and employment were found to play central roles in the multidimensional network, and aging was consistently associated with the risk of comorbidities. Further prospective population-based studies should be conducted to confirm these findings.

## Figures and Tables

**Table 1:** Demographic characteristics and dietary intake by blood pressure groups of study participants.

Factor	Normal (*n* = 2385)	Elevated (*n* = 2925)	Hypertension (*n* = 2113)	*p*-Value *
**Age (years)**	50.1 ± 7.5	52.2 ± 7.9	56.3 ± 7.8	**<0.001**
<50	1163 (48.8%)	1118 (38.2%)	430 (20.4%)	**<0.001**
50–54	592 (24.8%)	733 (25.1%)	447 (21.2%)	
55–59	352 (14.8%)	513 (17.5%)	447 (21.2%)	
≥60	278 (11.7%)	561 (19.2%)	789 (37.3%)	
**Sex**				
Male	1852 (77.7%)	1873 (64.0%)	1101 (52.1%)	**<0.001**
Female	533 (22.3%)	1052 (36.0%)	1012 (47.9%)	
**Marital status**				
Married, cohabitant	2032 (85.2%)	2510 (85.8%)	1813 (85.8%)	0.78
Others	353 (14.8%)	415 (14.2%)	300 (14.2%)	
**Education**				
<High school	217 (9.1%)	367 (12.5%)	373 (17.7%)	**<0.001**
High school graduate	863 (36.2%)	1098 (37.5%)	784 (37.1%)	
≥College	1305 (54.7%)	1460 (49.9%)	956 (45.2%)	
**Employment status**				
Employed	2261 (94.8%)	2695 (92.1%)	1847 (87.4%)	**<0.001**
Unemployed	124 (5.2%)	230 (7.9%)	266 (12.6%)	
**Monthly income (KRW)**				
<2 millions	423 (17.7%)	604 (20.6%)	565 (26.7%)	**<0.001**
2–4 millions	895 (37.5%)	1153 (39.4%)	864 (40.9%)	
≥4 millions	1067 (44.7%)	1168 (39.9%)	684 (32.4%)	
**Smoking**				
Never	1786 (74.9%)	1997 (68.3%)	1210 (57.3%)	**<0.001**
Past	340 (14.3%)	604 (20.6%)	647 (30.6%)	
Current	259 (10.9%)	324 (11.1%)	256 (12.1%)	
**Drinking**				
Never	966 (40.5%)	1178 (40.3%)	775 (36.7%)	**0.004**
Past	202 (8.5%)	201 (6.9%)	190 (9.0%)	
Current	1217 (51.0%)	1546 (52.9%)	1148 (54.3%)	
**Regular exercise**				
Yes	1075 (45.1%)	1352 (46.2%)	878 (41.6%)	**0.004**
No	1310 (54.9%)	1573 (53.8%)	1235 (58.4%)	
**Body mass index (kg/m^2^)**				
<23	1094 (45.9%)	1335 (45.6%)	962 (45.5%)	0.80
23–24.9	650 (27.3%)	763 (26.1%)	565 (26.7%)	
≥25	641 (26.9%)	827 (28.3%)	586 (27.7%)	
**Food group (g/day)**				
Cereals and grains	537.2 ± 212.4	578.1 ± 218.1	591.2 ± 214.3	**<0.001**
Potatoes and starches	41.8 ± 41.3	44.8 ± 45.0	43.9 ± 44.5	0.05
Sugars and sweets	4.7 ± 5.1	4.9 ± 5.1	4.6 ± 5.2	0.08
Legumes	55.5 ± 66.5	60.2 ± 65.8	60.6 ± 67.2	**0.01**
Seeds and nuts	5.7 ± 8.1	5.6 ± 9.8	5.8 ± 11.3	0.70
Vegetables	289.3 ± 188.1	315.8 ± 213.4	312.5 ± 202.1	**<0.001**
Mushrooms	8.7 ± 13.4	9.4 ± 13.9	9.4 ± 15.4	0.17
Fruits	226.2 ± 246.6	227.2 ± 262.5	211.3 ± 252.6	0.06
Meat and poultry	58.9 ± 52.8	60.7 ± 51.5	57.4 ± 53.0	0.08
Eggs	19.0 ± 19.4	18.4 ± 18.6	17.1 ± 18.1	**0.002**
Fishes and shellfishes	36.3 ± 32.7	39.4 ± 34.4	39.5 ± 33.0	**0.001**
Seaweeds	2.1 ± 2.2	2.3 ± 2.3	2.2 ± 2.4	**0.02**
Milks and dairy	108.1 ± 132.6	110.3 ± 134.0	105.5 ± 137.6	0.46
Oils and fats	3.5 ± 3.6	3.8 ± 3.8	3.5 ± 3.6	**0.01**
Beverages	66.9 ± 97.5	75.2 ± 115.5	71.2 ± 108.4	**0.02**
Seasonings	16.5 ± 13.9	17.5 ± 15.6	17.3 ± 14.5	**0.03**
**Dietary score**	558.0 ± 288.8	598.7 ± 321.9	588.9 ± 309.7	**<0.001**
Low (light eating)	884 (37.1%)	923 (31.6%)	667 (31.6%)	**<0.001**
Medium (normal eating)	779 (32.7%)	976 (33.4%)	719 (34.0%)	
High (heavy eating)	722 (30.3%)	1026 (35.1%)	727 (34.4%)	

Data presented as counts (percentages) for categorical variables and means ± standard deviations for continuous variables. * *p*-value from a Chi-square test for categorical variables and from ANOVA for continuous variables. Bold font indicates a significant difference.

**Table 2:** Demographic characteristics and dietary intake by total cholesterol groups of study participants.

Factor	Low (*n* = 1672)	Normal (*n* = 2280)	Borderline (*n* = 2576)	High (*n* = 895)	*p*-Value *
**Age (years)**	52.4 ± 8.2	52.0 ± 8.8	53.0 ± 7.7	53.9 ± 7.0	**<0.001**
<50	665 (39.8%)	977 (42.9%)	843 (32.7%)	226 (25.3%)	**<0.001**
50–54	366 (21.9%)	456 (20.0%)	679 (26.4%)	271 (30.3%)	
55–59	276 (16.5%)	324 (14.2%)	512 (19.9%)	200 (22.3%)	
≥60	365 (21.8%)	523 (22.9%)	542 (21.0%)	198 (22.1%)	
**Sex**					
Male	1030 (61.6%)	1424 (62.5%)	1718 (66.7%)	654 (73.1%)	**<0.001**
Female	642 (38.4%)	856 (37.5%)	858 (33.3%)	241 (26.9%)	
**Marital status**					
Married, cohabitant	1442 (86.2%)	1967 (86.3%)	2194 (85.2%)	752 (84.0%)	0.31
Others	230 (13.8%)	313 (13.7%)	382 (14.8%)	143 (16.0%)	
**Education**					
<High school	235 (14.1%)	272 (11.9%)	326 (12.7%)	124 (13.9%)	**0.002**
High school graduate	593 (35.5%)	837 (36.7%)	935 (36.3%)	380 (42.5%)	
≥College	844 (50.5%)	1171 (51.4%)	1315 (51.0%)	391 (43.7%)	
**Employment status**					
Employed	1504 (90.0%)	2087 (91.5%)	2381 (92.4%)	831 (92.8%)	**0.02**
Unemployed	168 (10.0%)	193 (8.5%)	195 (7.6%)	64 (7.2%)	
**Monthly income (KRW)**					
<2 millions	333 (19.9%)	498 (21.8%)	558 (21.7%)	203 (22.7%)	0.08
2–4 millions	643 (38.5%)	898 (39.4%)	995 (38.6%)	376 (42.0%)	
4 millions	696 (41.6%)	884 (38.8%)	1023 (39.7%)	316 (35.3%)	
**Smoking**					
Never	1088 (65.1%)	1488 (65.3%)	1781 (69.1%)	636 (71.1%)	**<0.001**
Past	387 (23.1%)	544 (23.9%)	512 (19.9%)	148 (16.5%)	
Current	197 (11.8%)	248 (10.9%)	283 (11.0%)	111 (12.4%)	
**Drinking**					
Never	638 (38.2%)	865 (37.9%)	1038 (40.3%)	378 (42.2%)	**0.01**
Past	127 (7.6%)	220 (9.6%)	189 (7.3%)	57 (6.4%)	
Current	907 (54.2%)	1195 (52.4%)	1349 (52.4%)	460 (51.4%)	
**Regular exercise**					
Yes	725 (43.4%)	1017 (44.6%)	1159 (45.0%)	404 (45.1%)	0.73
No	947 (56.6%)	1263 (55.4%)	1417 (55.0%)	491 (54.9%)	
**Body mass index (kg/m^2^)**					
<23	775 (46.4%)	1039 (45.6%)	1190 (46.2%)	387 (43.2%)	0.46
23–24.9	433 (25.9%)	614 (26.9%)	666 (25.9%)	265 (29.6%)	
≥25	464 (27.8%)	627 (27.5%)	720 (28.0%)	243 (27.2%)	
**Food group (g/day)**					
Cereals and grains	588.1 ± 219.6	577.2 ± 217.6	559.5 ± 212.1	537.6 ± 214.3	**<0.001**
Potatoes and starches	44.7 ± 44.7	44.8 ± 44.0	41.7 ± 41.8	44.2 ± 46.1	0.05
Sugars and sweets	4.8 ± 5.3	4.7 ± 5.1	4.8 ± 5.1	4.8 ± 5.0	0.76
Legumes	58.9 ± 63.0	58.1 ± 63.2	58.0 ± 67.6	62.8 ± 76.9	0.28
Seeds and nuts	5.3 ± 7.7	5.7 ± 10.7	5.5 ± 9.1	6.6 ± 12.4	**0.01**
Vegetables	309.3 ± 209.2	306.5 ± 196.3	303.8 ± 198.8	307.7 ± 216.9	0.85
Mushrooms	9.0 ± 14.0	9.3 ± 14.0	8.9 ± 13.1	9.9 ± 17.6	0.31
Fruits	219.6 ± 254.1	212.8 ± 251.6	228.3 ± 249.7	234.4 ± 276.8	0.08
Meat and poultry	59.6 ± 56.0	58.9 ± 50.6	58.7 ± 50.6	60.6 ± 54.7	0.80
Eggs	17.9 ± 18.5	17.5 ± 17.7	18.4 ± 18.3	20.6 ± 22.6	**<0.001**
Fishes and shellfishes	39.2 ± 35.3	37.5 ± 30.4	38.6 ± 33.3	39.0 ± 37.9	0.44
Seaweeds	2.2 ± 2.5	2.2 ± 2.2	2.1 ± 2.2	2.4 ± 2.6	0.07
Milks and dairy	107.3 ± 136.7	101.5 ± 128.9	112.4 ± 140.2	115.2 ± 127.7	**0.01**
Oils and fats	3.7 ± 3.8	3.5 ± 3.6	3.6 ± 3.6	3.7 ± 3.9	0.50
Beverages	70.3 ± 103.2	73.9 ± 110.8	71.7 ± 110.0	66.5 ± 103.5	0.36
Seasonings	17.2 ± 15.5	17.0 ± 14.3	17.0 ± 14.5	17.6 ± 15.3	0.72
**Dietary score**	588.0 ± 320.7	580.3 ± 299.2	579.7 ± 303.0	589.0 ± 324.7	0.74
Low (light eating)	536 (32.1%)	751 (32.9%)	875 (34.0%)	312 (34.9%)	0.35
Medium (normal eating)	554 (33.1%)	789 (34.6%)	855 (33.2%)	276 (30.8%)	
High (heavy eating)	582 (34.8%)	740 (32.5%)	846 (32.8%)	307 (34.3%)	

Data presented as counts (percentages) for categorical variables and means ± standard deviations for continuous variables. * *p*-value from a Chi-square test for categorical variables and from ANOVA for continuous variables. Bold font indicates a significant difference.

**Table 3:** Demographic characteristics and dietary intake by fasting glucose groups of study participants.

Factor	Normal (*n* = 6671)	Prediabetes and Diabetes (*n* = 752)	*p*-Value *
**Age (years)**	52.2 ± 8.0	57.0 ± 7.7	**<0.001**
<50	2576 (38.6%)	135 (18.0%)	**<0.001**
50–54	1622 (24.3%)	150 (19.9%)	
55–59	1157 (17.3%)	155 (20.6%)	
≥60	1316 (19.7%)	312 (41.5%)	
**Sex**			
Male	4513 (67.7%)	313 (41.6%)	**<0.001**
Female	2158 (32.3%)	439 (58.4%)	
**Marital status**			
Married, cohabitant	5708 (85.6%)	647 (86.0%)	0.77
Others	963 (14.4%)	105 (14.0%)	
**Education**			
<High school	843 (12.6%)	114 (15.2%)	0.12
High school graduate	2482 (37.2%)	263 (35.0%)	
≥College	3346 (50.2%)	375 (49.9%)	
**Employment status**			
Employed	6179 (92.6%)	624 (83.0%)	**<0.001**
Unemployed	492 (7.4%)	128 (17.0%)	
**Monthly income (KRW)**			
<2 millions	1380 (20.7%)	212 (28.2%)	**<0.001**
2–4 millions	2618 (39.2%)	294 (39.1%)	
≥4 millions	2673 (40.1%)	246 (32.7%)	
**Smoking**			
Never	4641 (69.6%)	352 (46.8%)	**<0.001**
Past	1319 (19.8%)	272 (36.2%)	
Current	711 (10.7%)	128 (17.0%)	
**Drinking**			
Never	2687 (40.3%)	232 (30.9%)	**<0.001**
Past	513 (7.7%)	80 (10.6%)	
Current	3471 (52.0%)	440 (58.5%)	
**Regular exercise**			
Yes	2996 (44.9%)	309 (41.1%)	0.05
No	3675 (55.1%)	443 (58.9%)	
**Body mass index (kg/m^2^)**			
<23	3039 (45.6%)	352 (46.8%)	0.63
23–24.9	1775 (26.6%)	203 (27.0%)	
≥25	1857 (27.8%)	197 (26.2%)	
**Food group (g/day)**			
Cereals and grains	565.9 ± 214.8	594.0 ± 228.1	**0.001**
Potatoes and starches	43.5 ± 43.3	44.5 ± 47.1	0.56
Sugars and sweets	4.8 ± 5.2	4.1 ± 4.6	**<0.001**
Legumes	58.3 ± 66.6	63.5 ± 64.8	**0.04**
Seeds and nuts	5.7 ± 9.8	5.7 ± 9.6	0.86
Vegetables	304.8 ± 202.2	320.1 ± 206.0	0.05
Mushrooms	9.2 ± 14.4	8.7 ± 11.8	0.25
Fruits	226.8 ± 257.9	182.4 ± 220.8	**<0.001**
Meat and poultry	59.1 ± 51.9	60.2 ± 56.7	0.60
Eggs	18.5 ± 18.9	16.4 ± 17.5	**0.003**
Fishes and shellfishes	38.1 ± 33.1	41.3 ± 36.6	**0.02**
Seaweeds	2.2 ± 2.3	2.3 ± 2.4	0.41
Milks and dairy	109.1 ± 135.5	100.3 ± 126.0	0.07
Oils and fats	3.7 ± 3.7	3.3 ± 3.4	**0.02**
Beverages	71.5 ± 107.9	70.9 ± 108.7	0.88
Seasonings	17.0 ± 14.7	18.5 ± 15.6	**0.01**
**Dietary score**	581.8 ± 308.8	592.3 ± 307.1	0.38
Low (light eating)	2218 (33.2%)	256 (34.0%)	0.05
Medium (normal eating)	2252 (33.8%)	222 (29.5%)	
High (heavy eating)	2201 (33.0%)	274 (36.4%)	

Data presented as counts (percentages) for categorical variables and means ± standard deviations for continuous variables. * *p*-value from a Chi-square test for categorical variables and from a *t*-test for continuous variables. Bold font indicates a significant difference.

**Table 4:** Demographic characteristics and dietary intake by glomerular filtration rate groups of study participants.

Factor	Normal (*n* = 1729)	Mildly Impairment (*n* = 5372)	Moderately Impairment (*n* = 322)	*p*-Value *
**Age (years)**	51.4 ± 7.4	52.6 ± 8.1	60.5 ± 8.0	**<0.001**
<50	700 (40.5%)	1973 (36.7%)	38 (11.8%)	**<0.001**
50–54	440 (25.4%)	1294 (24.1%)	38 (11.8%)	
55–59	332 (19.2%)	953 (17.7%)	27 (8.4%)	
≥60	257 (14.9%)	1152 (21.4%)	219 (68.0%)	
**Sex**				
Male	1159 (67.0%)	3449 (64.2%)	218 (67.7%)	0.06
Female	570 (33.0%)	1923 (35.8%)	104 (32.3%)	
**Marital status**				
Married, cohabitant	1476 (85.4%)	4607 (85.8%)	272 (84.5%)	0.77
Others	253 (14.6%)	765 (14.2%)	50 (15.5%)	
**Education**				
<High school	187 (10.8%)	709 (13.2%)	61 (18.9%)	**0.001**
High school graduate	638 (36.9%)	1994 (37.1%)	113 (35.1%)	
≥College	904 (52.3%)	2669 (49.7%)	148 (46.0%)	
**Employment status**				
Employed	1634 (94.5%)	4911 (91.4%)	258 (80.1%)	**<0.001**
Unemployed	95 (5.5%)	461 (8.6%)	64 (19.9%)	
**Monthly income (KRW)**				
<2 millions	304 (17.6%)	1181 (22.0%)	107 (33.2%)	**<0.001**
2–4 millions	669 (38.7%)	2119 (39.4%)	124 (38.5%)	
≥4 millions	756 (43.7%)	2072 (38.6%)	91 (28.3%)	
**Smoking**				
Never	1176 (68.0%)	3591 (66.8%)	226 (70.2%)	**0.001**
Past	337 (19.5%)	1174 (21.9%)	80 (24.8%)	
Current	216 (12.5%)	607 (11.3%)	16 (5.0%)	
**Drinking**				
Never	620 (35.9%)	2132 (39.7%)	167 (51.9%)	**<0.001**
Past	132 (7.6%)	424 (7.9%)	37 (11.5%)	
Current	977 (56.5%)	2816 (52.4%)	118 (36.6%)	
**Regular exercise**				
Yes	858 (49.6%)	2338 (43.5%)	109 (33.9%)	**<0.001**
No	871 (50.4%)	3034 (56.5%)	213 (66.1%)	
**Body mass index (kg/m^2^)**				
<23	783 (45.3%)	2444 (45.5%)	164 (50.9%)	0.34
23–24.9	458 (26.5%)	1447 (26.9%)	73 (22.7%)	
≥25	488 (28.2%)	1481 (27.6%)	85 (26.4%)	
**Food group (g/day)**	550.3 ± 222.1	575.1 ± 214.3	561.7 ± 212.5	**<0.001**
Cereals and grains	40.7 ± 41.9	44.0 ± 43.8	52.2 ± 50.4	**<0.001**
Potatoes and starches	4.4 ± 5.1	4.9 ± 5.2	4.2 ± 4.0	**<0.001**
Sugars and sweets	58.1 ± 72.4	58.5 ± 62.2	67.1 ± 95.2	0.07
Legumes	5.8 ± 8.6	5.6 ± 10.1	6.2 ± 10.2	0.56
Seeds and nuts	289.9 ± 182.4	310.6 ± 207.6	323.9 ± 216.7	**<0.001**
Vegetables	9.2 ± 13.8	9.1 ± 13.7	10.5 ± 21.9	0.20
Mushrooms	215.2 ± 253.7	224.3 ± 254.8	227.4 ± 258.7	0.41
Fruits	64.1 ± 57.8	58.2 ± 51.1	49.1 ± 39.6	**<0.001**
Meat and poultry	20.3 ± 20.1	17.7 ± 18.3	16.8 ± 18.2	**<0.001**
Eggs	35.0 ± 28.8	39.5 ± 34.6	39.1 ± 36.5	**<0.001**
Fishes and shellfishes	1.9 ± 1.9	2.2 ± 2.3	2.7 ± 3.5	**<0.001**
Seaweeds	102.0 ± 131.2	109.8 ± 134.6	115.3 ± 150.4	0.07
Milks and dairy	3.4 ± 3.7	3.7 ± 3.7	3.1 ± 3.2	**<0.001**
Oils and fats	59.8 ± 94.5	75.5 ± 111.7	65.7 ± 108.2	**<0.001**
Beverages	16.5 ± 14.5	17.2 ± 14.8	18.8 ± 15.6	**0.02**
Seasonings	557.0 ± 284.8	589.7 ± 314.1	608.5 ± 330.1	**<0.001**
**Dietary score**				
Low (light eating)	646 (37.4%)	1729 (32.2%)	99 (30.7%)	**0.001**
Medium (normal eating)	561 (32.4%)	1807 (33.6%)	106 (32.9%)	
High (heavy eating)	522 (30.2%)	1836 (34.2%)	117 (36.3%)	

Data presented as counts (percentages) for categorical variables and means ± standard deviations for continuous variables. * *p*-value from a Chi-square test for categorical variables and from ANOVA for continuous variables. Bold font indicates a significant difference.

**Table 5:** Association between demographic and dietary factors with comorbidity markers.

Factor	Blood Pressure (mmHg)				Total Cholesterol (mmol/L)						Fasting Glucose (mmol/L)		Glomerular Filtration Rate (mL/min/1.73 m^2^)			
	Elevated		Hypertension		Low		Borderline		High		Prediabetes and Diabetes		Mildly		Moderately	
	OR (95% CI)	*p*-Value	OR (95% CI)	*p*-Value	OR (95% CI)	*p*-Value	OR (95% CI)	*p*-Value	OR (95% CI)	*p*-Value	OR (95% CI)	*p*-Value	OR (95% CI)	*p*-Value	OR (95% CI)	*p*-Value
**Age (years)**																
<50	1.00		1.00		1.00		1.00		1.00		1.00		1.00		1.00	
50–54	**1.19** **(1.03–1.36)**	**0.02**	**1.82** **(1.53–2.16)**	**<0.001**	0.86 (0.73–1.03)	0.10	**1.58** **(1.33–1.86)**	**<0.001**	**2.41** **(1.93–3.02)**	**<0.001**	**1.71** **(1.33–2.18)**	**<0.001**	0.96 (0.84–1.11)	0.61	1.53 (0.95–2.44)	0.08
55–59	**1.29** **(1.09–1.53)**	**0.003**	**2.74** **(2.26–3.31)**	**<0.001**	0.82 (0.67–1.00)	0.05	**1.65** **(1.37–2.00)**	**<0.001**	**2.54** **(1.98–3.26)**	**<0.001**	**2.33** **(1.81–3.00)**	**<0.001**	0.87 (0.74–1.03)	0.10	1.38 (0.81–2.33)	0.23
≥60	**1.70** **(1.41–2.04)**	**<0.001**	**5.86** **(4.80–7.16)**	**<0.001**	1.03 (0.84–1.25)	0.80	**1.42** **(1.17–1.72)**	**<0.001**	**2.17** **(1.67–2.82)**	**<0.001**	**3.93** **(3.08–5.00)**	**<0.001**	**1.21** **(1.01–1.45)**	**0.04**	**12.5** **(8.28–19.0)**	**<0.001**
**Sex**																
Male	1.00		1.00		1.00		1.00		1.00		1.00		1.00		1.00	
Female	**2.52** **(2.08–3.04)**	**<0.001**	**2.94** **(2.38–3.63)**	**<0.001**	0.96 (0.79–1.19)	0.73	**0.78** **(0.64–0.96)**	**0.02**	**0.43** **(0.32–0.57)**	**<0.001**	**1.79** **(1.39–2.31)**	**<0.001**	1.19 (0.99–1.43)	0.06	**0.52** **(0.33–0.82)**	**0.01**
**Marital status**																
Married, cohabitant	1.00		1.00		1.00		1.00		1.00		1.00		1.00		1.00	
Others	1.03 (0.87–1.22)	0.73	1.00 (0.83–1.2)	0.96	0.95 (0.78–1.15)	0.58	1.03 (0.85–1.24)	0.76	1.02 (0.80–1.29)	0.89	1.05 (0.83–1.33)	0.66	0.91 (0.77–1.07)	0.24	0.75 (0.52–1.07)	0.11
**Education**																
<High school	1.00		1.00		1.00		1.00		1.00		1.00		1.00		1.00	
High school graduate	0.90 (0.73–1.09)	0.28	0.82 (0.67–1.02)	0.07	**1.26** **(1.02–1.56)**	**0.03**	**1.26** **(1.03–1.55)**	**0.03**	**1.42** **(1.09–1.84)**	**0.01**	1.11 (0.87–1.42)	0.40	0.92 (0.76–1.11)	0.39	1.01 (0.70–1.47)	0.95
≥College	**0.74** **(0.61–0.91)**	**0.003**	**0.62** **(0.50–0.77)**	**<0.001**	**1.28** **(1.03–1.58)**	**0.02**	**1.42** **(1.16–1.75)**	**0.001**	1.31 (1.00–1.72)	0.05	1.08 (0.85–1.39)	0.52	0.87 (0.72–1.06)	0.16	1.07 (0.73–1.56)	0.73
**Employment status**																
Employed	1.00		1.00		1.00		1.00		1.00		1.00		1.00		1.00	
Unemployed	0.99 (0.77–1.27)	0.92	0.83 (0.64–1.07)	0.15	**0.75** **(0.58–0.95)**	**0.02**	**0.73** **(0.58–0.93)**	**0.01**	0.75 (0.54–1.05)	0.09	1.04 (0.81–1.32)	0.77	1.28 (1.00–1.63)	0.05	**1.79** **(1.20–2.66)**	**0.004**
**Monthly income (KRW)**																
<2 millions	1.00		1.00		1.00		1.00		1.00		1.00		1.00		1.00	
2–4 millions	0.97 (0.82–1.14)	0.72	0.92 (0.77–1.09)	0.34	0.88 (0.73–1.05)	0.16	0.91 (0.76–1.08)	0.27	1.01 (0.81–1.27)	0.91	0.84 (0.68–1.02)	0.08	0.85 (0.72–1.00)	0.05	0.80 (0.58–1.10)	0.16
≥ 4 millions	0.92 (0.77–1.09)	0.32	**0.80** **(0.66–0.96)**	**0.02**	**0.77** **(0.64–0.94)**	**0.01**	0.83 (0.69–1.01)	0.06	0.81 (0.63–1.04)	0.10	0.83 (0.66–1.04)	0.11	**0.76** **(0.64–0.90)**	**0.002**	0.75 (0.53–1.08)	0.12
**Smoking**																
Never	1.00		1.00		1.00		1.00		1.00		1.00		1.00		1.00	
Past	0.85 (0.69–1.04)	0.12	1.16 (0.93–1.46)	0.18	1.07 (0.86–1.33)	0.54	0.92 (0.74–1.15)	0.48	1.12 (0.83–1.52)	0.46	**1.40** **(1.09–1.81)**	**0.01**	1.02 (0.84–1.24)	0.81	1.37 (0.87–2.16)	0.18
Current	**0.60** **(0.48–0.76)**	**<0.001**	**0.75** **(0.58–0.97)**	**0.03**	0.95 (0.74–1.23)	0.72	1.02 (0.80–1.31)	0.87	**1.72** **(1.24–2.40)**	**0.001**	**1.63** **(1.22–2.18)**	**0.001**	0.90 (0.72–1.11)	0.33	0.79 (0.42–1.48)	0.46
**Drinking**																
Never	1.00		1.00		1.00		1.00		1.00		1.00		1.00		1.00	
Past	**0.69** **(0.55–0.87)**	**0.001**	0.84 (0.65–1.08)	0.17	1.27 (0.99–1.64)	0.06	1.07 (0.83–1.39)	0.59	1.05 (0.74–1.49)	0.79	1.21 (0.89–1.63)	0.22	0.86 (0.69–1.07)	0.18	1.22 (0.77–1.94)	0.39
Current	0.97 (0.85–1.10)	0.59	1.11 (0.95–1.28)	0.19	0.98 (0.84–1.14)	0.80	1.07 (0.92–1.24)	0.37	1.18 (0.97–1.43)	0.09	1.17 (0.96–1.42)	0.13	**0.82** **(0.72–0.93)**	**0.003**	**0.71** **(0.52–0.96)**	**0.03**
**Regular exercise**																
Yes	1.00		1.00		1.00		1.00		1.00		1.00		1.00		1.00	
No	**0.88** **(0.79–0.98)**	**0.03**	0.95 (0.83–1.08)	0.41	0.97 (0.86–1.11)	0.70	0.94 (0.82–1.06)	0.31	0.93 (0.79–1.1)	0.40	1.00 (0.85–1.17)	0.98	**1.24** **(1.11–1.38)**	**<0.001**	**1.41** **(1.09–1.84)**	**0.01**
**Body mass index (kg/m^2^)**																
<23	1.00		1.00		1.00		1.00		1.00		1.00		1.00		1.00	
23–24.9	0.97 (0.85–1.12)	0.72	1.04 (0.89–1.21)	0.63	1.05 (0.90–1.23)	0.50	1.00 (0.86–1.16)	0.99	1.22 (1.00–1.49)	0.05	1.02 (0.85–1.23)	0.83	1.02 (0.89–1.17)	0.76	0.80 (0.58–1.09)	0.15
≥25	1.06 (0.93–1.21)	0.37	1.07 (0.92–1.24)	0.38	1.01 (0.87–1.17)	0.91	1.01 (0.87–1.18)	0.86	1.05 (0.86–1.28)	0.66	0.93 (0.77–1.13)	0.47	0.97 (0.85–1.11)	0.66	0.83 (0.62–1.12)	0.22
**Dietary score**																
Low (light eating)	1.00		1.00		1.00		1.00		1.00		1.00		1.00		1.00	
Medium (normal eating)	**1.23** **(1.07–1.40)**	**0.003**	**1.26** **(1.08–1.47)**	**0.003**	1.02 (0.87–1.19)	0.79	0.94 (0.81–1.09)	0.42	0.85 (0.69–1.04)	0.11	0.84 (0.69–1.02)	0.09	**1.21** **(1.06–1.38)**	**0.01**	1.20 (0.88–1.63)	0.25
High (heavy eating)	**1.40** **(1.22–1.60)**	**<0.001**	**1.36** **(1.16–1.58)**	**<.001**	0.91 (0.78–1.07)	0.25	0.87 (0.75–1.02)	0.08	0.86 (0.71–1.06)	0.15	1.10 (0.91–1.33)	0.32	**1.29** **(1.13–1.48)**	**<0.001**	1.29 (0.95–1.75)	0.11

The association between each dietary or demographic factor and comorbidity marker was adjusted for the remaining factors. Bold font indicates significant difference.

## Data Availability

The data are available from the corresponding author upon reasonable request.

## Supplementary Materials

The following are available online at https://www.mdpi.com/article/10.3390/nu13103563/s1. Supplementary Figure S1: Bootstrapped confidence intervals for the edge weight accuracy, Figure S2: Stability of the centrality indices of the mixed graphical model-identified network, Figure S3: Bootstrapped difference test between nonzero edge weights, Figure S4: Bootstrapped difference test between node strength, Table S1: Adjacency matrix of pairwise correlation of dietary intake of 16 food groups, Table S2: Adjacency matrix of pairwise interaction of demographics, dietary intake, and comorbidity markers, Table S3: Centrality indices and prediction estimates of nodes in the network of demographics, dietary intake, and comorbidity markers, Table S4: Adjacency matrix of pairwise interaction of demographics, food groups, and comorbidity markers, Table S5: Centrality indices and prediction estimates of nodes in the network of demographics, dietary intake, and comorbidity markers, Table S6: Adjacency matrix of pairwise interaction of demographics, dietary intake, and comorbidity marker, Table S7: Centrality indices and prediction estimates of nodes in the network of modifiable demographics, dietary intake, and comorbidity markers.
