# Supplementary material for: Network Analysis of Demographics, Dietary Intake, and Comorbidity Interactions
Source: Nutrients. 2021 Oct 12;13(10):3563. doi: 10.3390/nu13103563 (PMC8539503; doi:10.3390/nu13103563)
Supplement: Supplementary file 1 [file nutrients-13-03563-s001.zip › nutrients-1357578-supplementary.pdf]

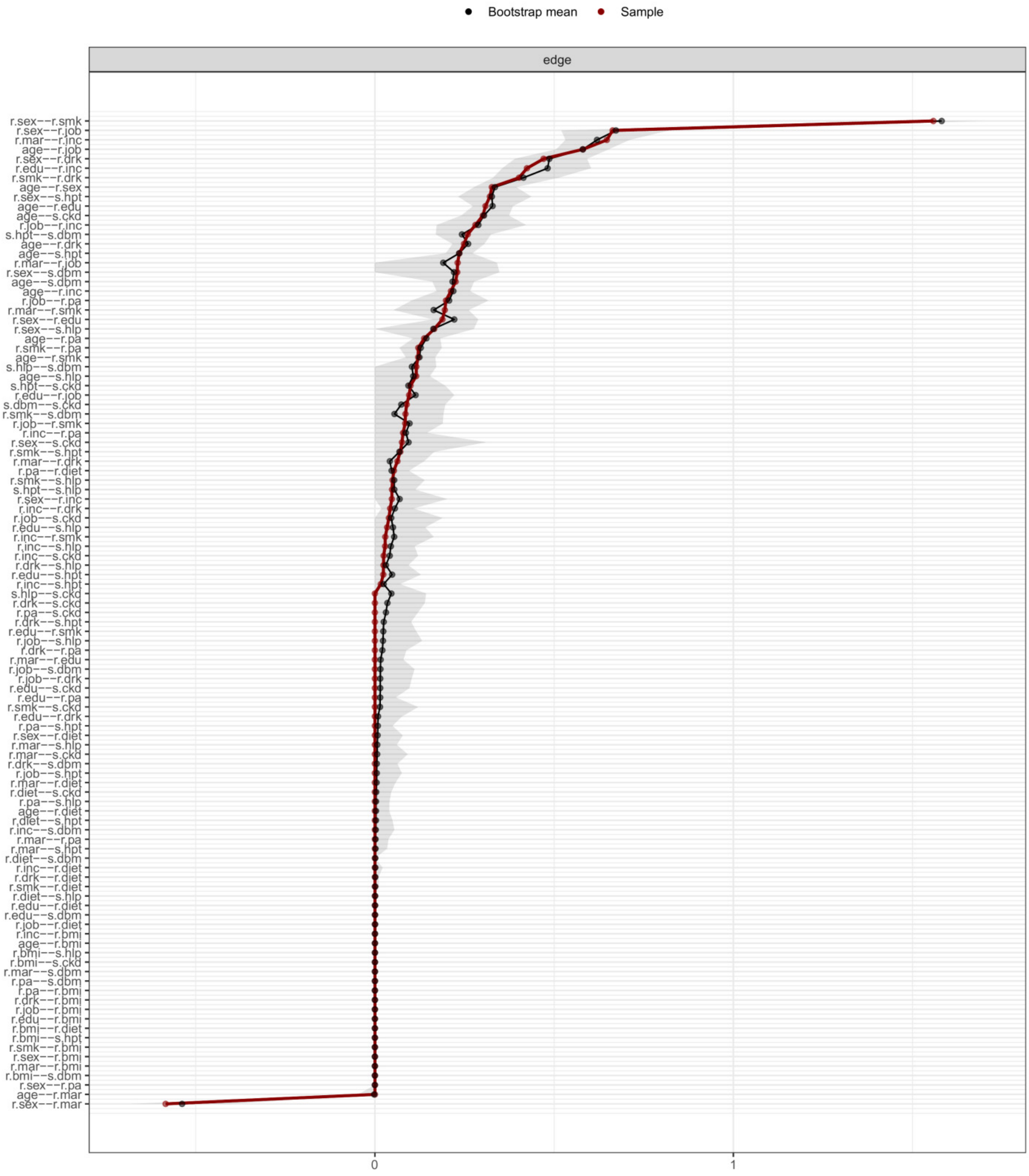

Figure S1. Bootstrapped confidence intervals for the edge weight accuracy. r.sex: sex, r.mar: marital status, r.edu: educational level, r.job: employment, r.inc: monthly income, r.smk: smoking, r.drk: alcohol consumption, r.pa: regular exercise, r.bmi: body mass index, r.diet: dietary score, s.hpt: blood pressure, s.hlp: total cholesterol, s.dbm: fasting glucose, s.ckd: glomerular filtration rate.

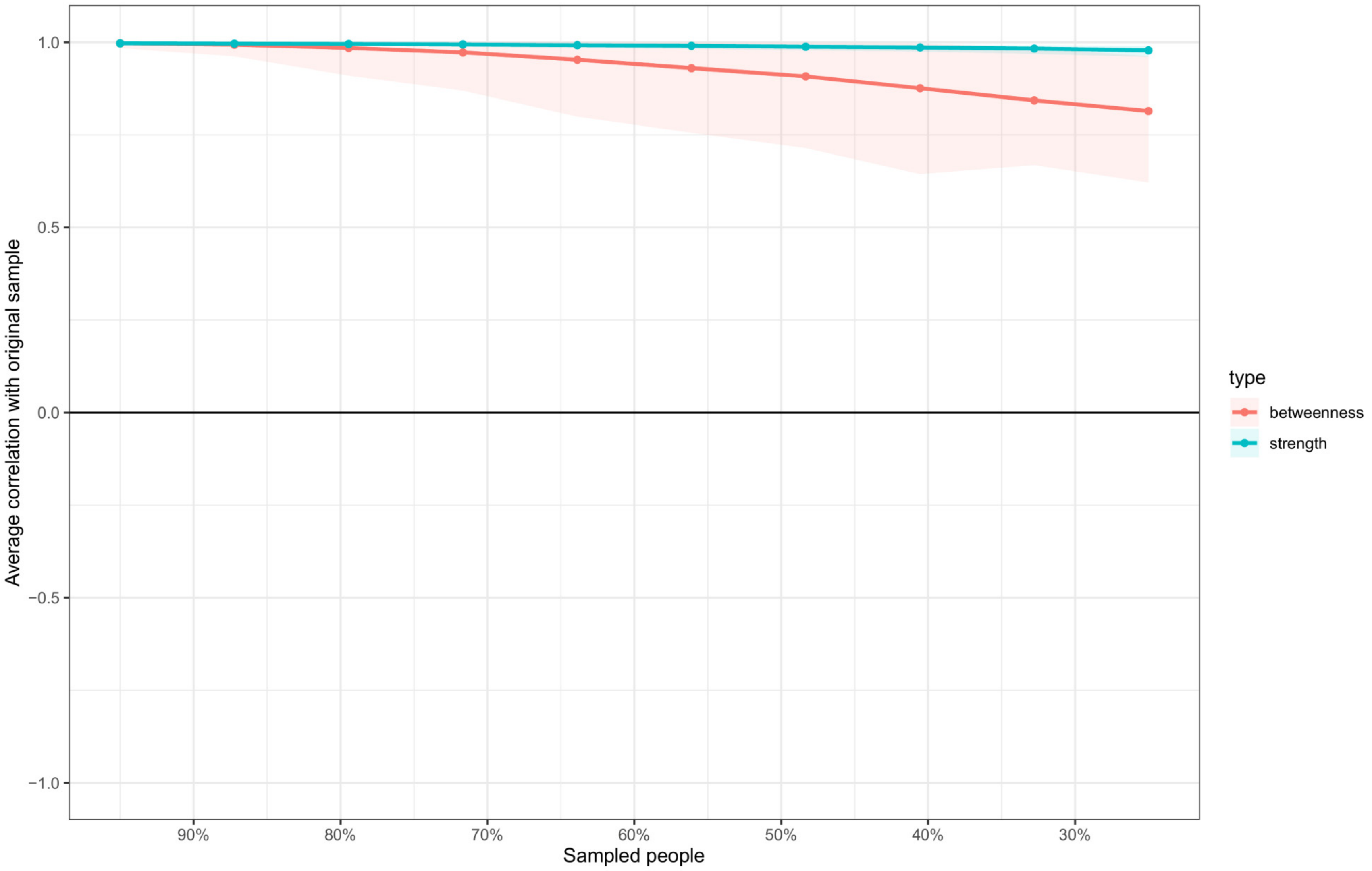

Figure S2. Stability of the centrality indices of the mixed graphical model-identified network.

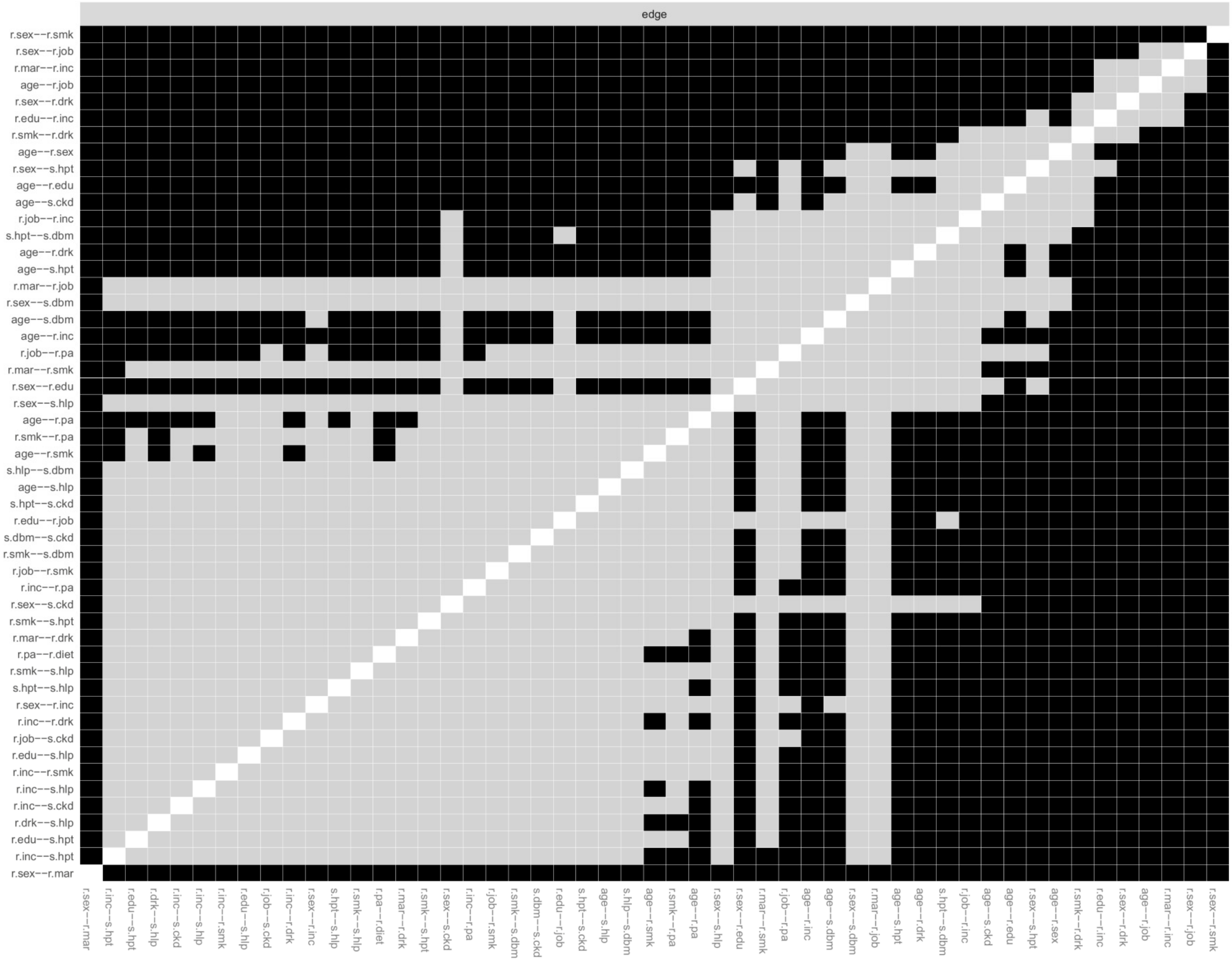

Figure S3. Bootstrapped difference test between nonzero edge weights. Gray boxes indicate edges that are not significantly different from one another, and black boxes indicate edges that are significantly different from one another. r.sex: sex, r.mar: marital status, r.edu: educational level, r.job: employment, r.inc: monthly income, r.smk: smoking, r.drk: alcohol consumption, r.pa: regular exercise, r.bmi: body mass index, r.diet: dietary score, s.hpt: blood pressure, s.hlp: total cholesterol, s.dbm: fasting glucose, s.ckd: glomerular filtration rate.

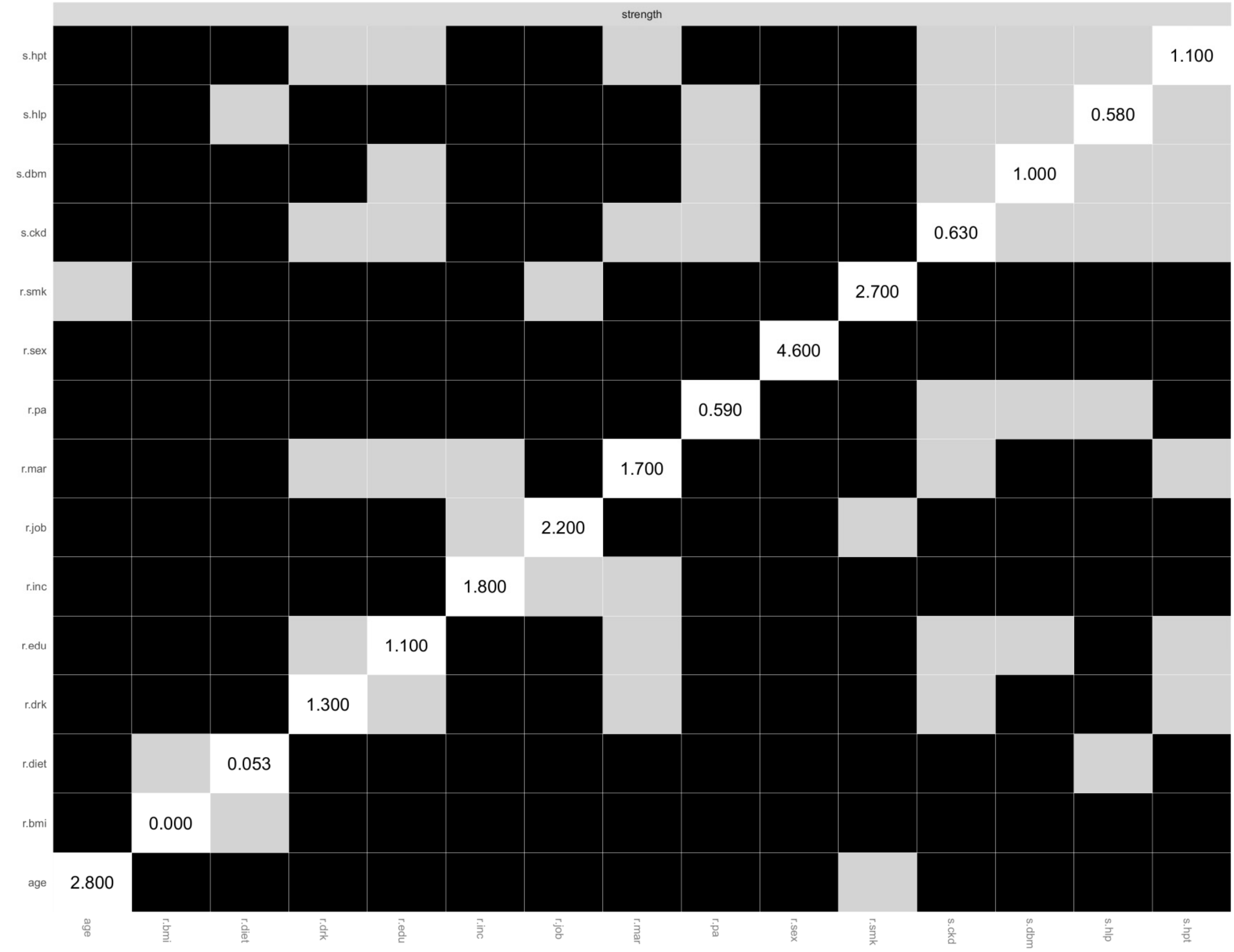

Figure S4. Bootstrapped difference test between node strengths. Gray boxes indicate nodes that are not significantly different from one another, and black boxes indicate nodes that are significantly different from one another. r.sex: sex, r.mar: marital status, r.edu: educational level, r.job: employment, r.inc: monthly income, r.smk: smoking, r.drk: alcohol consumption, r.pa: regular exercise, r.bmi: body mass index, r.diet: dietary score, s.hpt: blood pressure, s.hlp: total cholesterol, s.dbm: fasting glucose, s.ckd: glomerular filtration rate.

Table S1. Adjacency matrix for pairwise correlation of dietary intake of 16 food groups.

	Cereals and grains	Potatoes and starches	Sugars and sweets	Legumes	Seeds and nuts	Vegetables	Mushrooms	Fruits	Meat and poultry	Eggs	Fishes and shellfishes	Seaweeds	Milks and dairy	Oils and fats	Beverages	Seasonings
**Cereals and grains**	–															
**Potatoes and starches**		–														
**Sugars and sweets**		0.04	–													
**Legumes**		0.18		–												
**Seeds and nuts**	−0.07			0.07	–											
**Vegetables**	0.08	0.06				–										
**Mushrooms**	−0.05		−0.06		0.10	0.28	–									
**Fruits**	−0.09	−0.06		−0.06		0.08		–								
**Meat and poultry**	0.09	−0.06	−0.05	−0.06		0.08	0.05	−0.05	–							
**Eggs**		0.08		0.08					0.13	–						
**Fishes and shellfishes**		0.07		0.06	0.05	0.13	0.16		0.21		–					
**Seaweeds**		0.05		0.10		0.21		0.06			0.20	–				
**Milks and dairy**	−0.09		0.04	0.05				0.12		0.08		0.07	–			
**Oils and fats**	0.09		0.67				0.06	−0.06	0.21	0.05		−0.04	−0.05	–		
**Beverages**			0.04			0.04					0.09				–	
**Seasonings**		0.33		0.14	0.10	0.42	−0.10	0.24	0.04		0.06			0.06		–

Table S2. Adjacency matrix for pairwise interaction of demographics, dietary intake, and comorbidity markers.

	Age	Sex	Marital Status	Educational Level	Employment	Monthly Income	Smoking	Drinking	Regular Exercise	Body Mass Index	Dietary Score	Blood Pressure	Total Cholesterol	Fasting Glucose	Glomerular Filtration rate
**Age**	-														
**Sex**	0.33	-													
**Marriage**		0.58	-												
**Education**	0.31	0.19		-											
**Employment**	0.58	0.66	0.23	0.10	-										
**Income**	0.21	0.05	0.65	0.42	0.28	-									
**Smoking**	0.12	1.56	0.20		0.08	0.03	-								
**Drinking**	0.25	0.47	0.06			0.04	0.40	-							
**Regular exercise**	0.14				0.20	0.08	0.12		-						
**Body mass index**										-					

**Dietary score**									0.05		-				
**Blood pressure**	0.24	0.32		0.02		0.02	0.07					-			
**Total cholesterol**	0.12	0.17		0.03		0.03	0.05	0.02				0.05	-		
**Fasting glucose**	0.23	0.23					0.09					0.26	0.12	-	
**Glomerular filtration rate**	0.30	0.08			0.04	0.02						0.10		0.09	-

Table S3. Centrality indices and prediction estimates of nodes in the network of demographics, dietary intake, and comorbidity markers.

Node	Strength	Closeness	Betweenness	Explained Variance (%) *	Correct Classification (%) ¶
Age	2.81	0.013	18	33.1	-
Sex	4.63	0.014	41	-	89.4
Marriage	1.72	0.012	6	-	85.7
Education	1.07	0.009	0	-	56.6
Employment	2.17	0.014	22	-	92.3
Income	1.83	0.011	1	-	52.0
Smoking	2.72	0.013	0	-	79.9
Drinking	1.25	0.011	0	-	65.7
Exercise	0.59	0.008	12	-	58.9
Body mass index	0	-	0	-	45.7
Dietary score	0.05	0.003	0	-	36.8
Blood pressure	1.07	0.010	0	-	47.5
Total cholesterol	0.58	0.007	0	-	38.6
Fasting glucose	1.00	0.009	0	-	89.9
Glomerular filtration rate	0.63	0.008	0	-	72.4

* Error function for continuous variables. ¶ Error function for categorical variables.

Table S4. Adjacency matrix for pairwise interaction of demographics, food groups, and comorbidity markers.

	Age	Sex	Marriage	Education	Employment	Income	Smoking	Drinking	Regular Exercise	Body Mass Index	Cereals and Grains	Potatoes and Starches	Sugars and Sweets	Legumes	Seeds and Nuts	Vegetables	Mushrooms	Fruits	Meat and Poultry	Eggs	Fishes and Shellfishes	Seaweeds	Milks and Dairy	Oils and Fats	Beverages	Seasonings	Blood pressure	Total cholesterol	Fasting glucose	Glomerular Filtration rate
**Age**	-																													
**Sex**	0.38	-																												
**Marriage**		0.46	-																											
**Education**	0.30	0.23		-																										
**Employment**	0.56	0.70	0.17	0.09	-																									

**Income**	0.20	0.07	0.55	0.35	0.25	-																								
**Smoking**	0.10	1.53	0.14		0.08		-																							
**Drinking**	0.23	0.48				0.03	0.38	-																						
**Regular exercise**	0.12				0.21	0.06	0.11	0.02	-																					
**Body mass index**										-																				
**Cereals and grains**		0.40	0.02	0.03		0.03			0.03		-																			
**Potatoes and starches**		0.10										-																		
**Sugars and sweets**	0.03												-																	
**Legumes**												0.17		-																
**Seeds and nuts**									0.05		0.02			0.05	-															
**Vegetables**	0.02			0.02							0.05	0.05				-														
**Mushrooms**	0.03	0.08													0.08	0.26	-													
**Fruits**		0.23		0.03		0.04	0.03	0.03	0.04		0.02					0.07		-												
**Meat and poultry**	0.11	0.12				0.02	0.02	0.07			0.05					0.09	0.03		-											
**Eggs**				0.05								0.06		0.06	0.03		0.02		0.11	-										
**Fishes and shellfishes**												0.05		0.05	0.04	0.14	0.14		0.18		-									
**Seaweeds**		0.11										0.03		0.09		0.22		0.03			0.18	-								
**Milks and dairy**									0.08		0.04			0.03	0.03			0.10		0.06		0.06	-							
**Oils and fats**	0.04						0.06				0.07		0.63						0.17					-						
**Beverages**		0.07														0.04		0.03	0.02		0.07		0.02		-					
**Seasonings**												0.33		0.13	0.10	0.42		0.23			0.06					-				
**Blood pressure**	0.23	0.30		0.01			0.02																				-			
**Total cholesterol**	0.07	0.07		0.01			0.01				0.02									0.01							0.02	-		
**Fasting glucose**	0.21	0.26					0.04																				0.21	0.08	-	
**Glomerular filtration rate**	0.29				0.04				0.03		0.01								0.04	0.04					0.03		0.04		0.06	-

Table S5. Centrality indices and prediction estimates of nodes in the network of demographics, dietary intake, and comorbidity markers.

Node	Strength	Closeness	Betweenness	Explained Variance (%) *	Correct Classification (%) ¶
Age	2.92	0.004	34	35.5	
Sex	5.60	0.004	235		90.2
Marriage	1.34	0.003	22		85.6
Education	1.12	0.003	0		56.9
Employment	2.10	0.004	25		92.3
Income	1.60	0.003	1		51.6
Smoking	2.52	0.004	0		80.2
Drinking	1.23	0.003	0		66.5
Regular exercise	0.74	0.003	0		61.7
Body mass index	0	NA	0		45.7
Cereals and grains	0.8	0.003	0	12.6	
Potatoes and starches	0.78	0.003	16	38.1	
Sugars and sweets	0.66	0.002	0	47.7	
Legumes	0.59	0.002	0	25.4	
Seeds and nuts	0.39	0.002	0	14.0	
Vegetables	1.39	0.003	28	59.9	
Mushrooms	0.65	0.002	0	26.1	
Fruits	0.88	0.003	64	24.2	
Meat and poultry	1.02	0.003	83	26.9	
Eggs	0.45	0.002	0	12.7	
Fishes and shellfishes	0.92	0.002	18	39.3	
Seaweeds	0.72	0.003	3	30.6	
Milks and dairy	0.42	0.002	0	9.7	
Oils and fats	0.97	0.002	27	52.7	
Beverages	0.29	0.002	0	7.2	
Seasonings	1.27	0.003	61	59.3	
Blood pressure	0.83	0.003	0		47.3
Total cholesterol	0.30	0.002	0		38.6
Fasting glucose	0.86	0.003	0		89.9
Glomerular filtration rate	0.57	0.003	0		72.4

* Error function for continuous variables. ¶ Error function for categorical variables.

Table S6. Adjacency matrix for pairwise interaction of demographics, dietary intake, and comorbidity markers.

	Marital Status	Educational Level	Employment	Monthly Income	Smoking	Drinking	Regular Exercise	Body Mass Index	Dietary Score	Blood Pressure	Total Cholesterol	Fasting Glucose	Glomerular Filtration RATE
**Marriage**	-												
**Education**		-											
**Employment**	0.06	0.11	-										
**Income**	0.62	0.57	0.48	-									
**Smoking**	0.13	0.09	0.49	0.06	-								
**Drinking**		0.03		0.07	0.67	-							

**Regular exercise**			0.29	0.05	0.13		-						
**Body mass index**								-					
**Dietary score**							0.05		-				
**Blood pressure**		0.13	0.10	0.08	0.20					-			
**Total cholesterol**										0.04	-		
**Fasting glucose**			0.19		0.24					0.35	0.06	-	
**Glomerular filtration rate**			0.22	0.07		0.06				0.19		0.15	-

Table S7. Centrality indices and prediction estimates of nodes in the network of modifiable demographics, dietary intake, and comorbidity markers.

**Node**	**Strength**	**Closeness**	**Betweenness**	**Correct Classification (%) ***
Marriage	0.81	0.009	0	85.6
Education	0.94	0.009	0	54.9
Employment	1.94	0.012	37	91.6
Income	2.01	0.011	18	51.9
Smoking	2.01	0.012	15	70.6
Drinking	0.83	0.010	0	61.6
Regular exercise	0.52	0.009	10	57.1
Body mass index	0	NA	0	45.7
Dietary score	0.05	0.003	0	36.8
Blood pressure	1.08	0.009	0	45.3
Total cholesterol	0.10	0.004	0	36.2
Fasting glucose	0.99	0.010	10	89.9
Glomerular filtration rate	0.70	0.009	0	72.4

* Error function for categorical variables.
